# Characterization of test positivity among patients with coronavirus disease 2019 (COVID-19) in three electronic health records databases, February–November 2020

**DOI:** 10.1186/s12889-022-13635-6

**Published:** 2022-06-18

**Authors:** Patrick Saunders-Hastings, Cindy Ke Zhou, Shayan Hobbi, Hui-Lee Wong, Patricia Lloyd, Eva Boyd, Nader Alawar, Tainya C. Clarke, Jeff Beers, Timothy Burrell, Azadeh Shoaibi

**Affiliations:** 1 Gevity Consulting Inc., part of Accenture, Ottawa, ON Canada; 2 Center for Biologics Evaluation and Research, US Food and Drug Administration, Silver Spring, MD USA; 3 IBM Consulting, Bethesda, MD USA; 4 IBM Watson Health, Cambridge, MA USA

**Keywords:** COVID-19, Test positivity, EHR

## Abstract

**Background:**

Monitoring COVID-19 testing volumes and test positivity is an integral part of the response to the pandemic. We described the characteristics of individuals who were tested and tested positive for SARS-CoV-2 during the pre-vaccine phase of the pandemic in the United States (U.S.).

**Methods:**

This descriptive study analyzed three U.S. electronic health record (EHR) databases (Explorys, Academic Health System, and OneFlorida) between February and November 2020, identifying patients who received an interpretable nucleic acid amplification test (NAAT) result. Test-level data were used to characterize the settings in which tests were administered. Patient-level data were used to calculate test positivity rates and characterize the demographics, comorbidities, and hospitalization rates of COVID-19-positive patients.

**Results:**

Over 40% of tests were conducted in outpatient care settings, with a median time between test order and result of 0–1 day for most settings. Patients tested were mostly female (55.6–57.7%), 18–44 years of age (33.9–41.2%), and Caucasian (44.0–66.7%). The overall test positivity rate was 13.0% in Explorys, 8.0% in Academic Health System, and 8.9% in OneFlorida. The proportion of patients hospitalized within 14 days of a positive COVID-19 NAAT result was 24.2–33.1% across databases, with patients over 75 years demonstrating the highest hospitalization rates (46.7–69.7% of positive tests).

**Conclusions:**

This analysis of COVID-19 testing volume and positivity patterns across three large EHR databases provides insight into the characteristics of COVID-19-tested, COVID-19-test-positive, and hospitalized COVID-19-test-positive patients during the early phase of the pandemic in the U.S.

**Supplementary Information:**

The online version contains supplementary material available at 10.1186/s12889-022-13635-6.

## Background

From the beginning of the COVID-19 pandemic to December 31, 2021, there have been over 55 million confirmed cases of COVID-19 in the United States (U.S.), leading to more than 800,000 documented deaths [1]. Since February 2020, two types of diagnostic tests have received emergency use authorization (EUA) from the U.S. Food and Drug Administration (FDA) Center for Devices and Radiological Health [2]. The most accurate way to confirm a COVID-19 diagnosis is via nucleic acid amplification test (NAAT)-based assays, or more specifically, reverse-transcription polymerase chain reaction (RT-PCR) assays, to detect viral RNA. Meanwhile, antigen tests are based on the detection of viral proteins but are less sensitive than molecular tests [3].

Testing has been an integral part of the response to COVID-19, as case detection underlies many other pandemic response activities, including contact tracing and isolation [4, 5]. It is also essential for understanding the extent of transmission in a given community or geographic area [4]. For example, determining the proportion of positive tests, known as the test positivity rate, is an important indicator that can help with real-time monitoring of disease transmission in a specific geographic area, provide early warning of increasing transmission, and inform the overall prevention and control strategy for COVID-19 [4]. In addition, test positivity has also been used as a proxy for COVID-19 circulation in order to adjust estimates of vaccine effectiveness against symptomatic COVID-19 infection [6].

The FDA Center for Biologics Evaluation and Research (CBER) has a mission to conduct policy and regulatory reviews of biologics and related products, including blood products, vaccines, allergenics, tissues, and cellular and gene therapies. The Biologics Effectiveness and Safety (BEST) Initiative is a program initiated by CBER with the objective of assessing the safety and effectiveness of biologic products using large datasets of heathcare data. Pandemic preparedness and response are priorities for the CBER BEST Initiative. As an active surveillance program, BEST contributes to CBER’s mission to evaluate and ensure the safety and effectiveness of biologic products, including vaccines. Data from the three electronic health record (EHR) data sources participating in the BEST network were leveraged to describe the testing patterns, the characteristics of individuals who received testing and tested positive for severe acute respiratory syndrome coronavirus 2 (SARS-CoV-2), the temporal trends in test positivity, and the hospitalization rates among test-positive cases during the pre-vaccine phase of the COVID-19 pandemic between February and November 2020.

## Methods

### Study population

We conducted a retrospective study of individuals who received at least one interpretable SARS-CoV-2 NAAT result during the study period of February 5–November 30, 2020, in three separate EHR databases (Explorys, Academic Health System, and OneFlorida) spanning across six different states in the eastern U.S. and providing health care services to approximately 78 million patients per year. Explorys contained data on health encounters mostly from Ohio, Louisiana, Georgia, Florida, and New York. Meanwhile, OneFlorida contained data on health encounters from Florida and Academic Health System encounters were primarily from the Mid-Atlantic region. The study start date was chosen based on the first EUA of SARS-CoV-2 NAAT tests for COVID-19 diagnosis (February 5, 2020) [2]. However, testing data were not observed in the study databases until March 2020, therefore data are presented for March–November 2020. The study period end date represents the last full month of data available before FDA granted EUA to the first COVID-19 vaccine on December 11, 2020.

### Patient consent statement

This study does not include factors necessitating patient consent.

### Test-level analyses

The number of interpretable NAATs conducted (testing volume) was summarized, overall and by the healthcare setting in which the test was ordered. Tests were identified by Logical Observation Identifiers Names and Codes (LOINC) codes (Supplementary Material, Appendix A). An “interpretable test result” was defined as a non-null result indicating the presence or absence of SARS-CoV-2 ribonucleic acid (RNA) above or below the specified threshold of the test, respectively (i.e., positive, or negative results). Uninterpretable test results were defined as records without a value in the result (i.e., erroneous or duplicate null test result records), result entries that indicated the test was not performed, inconclusive results due to faulty samples, or otherwise non-positive or non-negative duplicate results that included a note to refer to a different valid test result record. Based on a preliminary query, uninterpretable test results represented about 2–3% of all COVID-19 test result records. Uninterpretable test results were not considered in the denominator for test positivity rate calculations as they artificially deflate the positivity rate.

Healthcare settings were categorized into the following groups for reporting purposes: outpatient/clinic, telehealth, inpatient, emergency room (ER), long-term care, other, and unknown. To understand the turnaround time (lag) between ordering a NAAT test and receipt of testing results by the healthcare provider, the number of days between the test order and test results were summarized using the median (5th and 95th percentiles) overall, by healthcare setting, and by calendar month.

### Patient-level analyses

Patient-level data were used to calculate the test positivity rate defined as the proportion of patients with a positive SARS-CoV-2 NAAT result, among those who received at least one interpretable SARS-CoV-2 NAAT result. The positivity rates were stratified by the healthcare setting of the test order (where available) and the patient’s baseline demographics and comorbidities. Baseline was defined as the period of 365 to 14 days prior to a patient’s first test result. Demographics included sex, age groups, and self-reported race and ethnicity. As a sensitivity analysis, test positivity rates were stratified by race, with the patients that identified as ‘Other’ or ‘Unknown’ added to the numerator and denominator of each of the other race groups. Comorbidities were characterized using the Charlson Comorbidity Index (CCI) [7].

We described the characteristics of the tested patient population in each EHR network using demographic and comorbidity data for patients who received a COVID-19 NAAT. This population was compared to patients with at least one positive COVID-19 NAAT.

Hospitalization status was determined by identifying an inpatient admission within ±14 days from the date of their first positive NAAT. Hospitalization rates were computed overall for each EHR database and stratified by age, sex, race, and ethnicity. ER admission was identified by capturing patients who were admitted to an ER within ±14 days from the date of their first positive NAAT. Patients who were hospitalized could also have been admitted to an emergency room, and vice versa, as these groups were not mutually exclusive. For hospitalized patients, the prevalence of comorbidities was determined using a lookback period defined as 379 days to 14 days prior to the patient’s first inpatient admission date, or — if the patient was not hospitalized — the lookback period was defined as 379 to 14 days prior to the patient’s first interpretable NAAT.

## Results

### Test-level analyses

Between February 5 and November 30, 2020, 1,303,953, 232,044, and 534,525 interpretable NAATs were recorded in Explorys, Academic Health System, and OneFlorida, respectively.

Over 40% of tests were conducted in outpatient care settings across each EHR network. The median time between test order and result was generally 0–1 day, with long-term care (median 10 days, 5th–95th percentile: 0–39 days) and telehealth (median 2 days, 5th–95th percentile: 0–17 days) orders in Explorys being the only exceptions. Additional data on the distribution of tests and test turnaround time by healthcare setting are presented in Table 1**.** Meanwhile, data on test turnaround time by month are presented in Appendix B (Table B1), Turnaround time varied by month, but never exceed a median of 2 days, as observed in Explorys and Academic Health System in March 2020.

**Table 1:** Number of tests and median test turnaround time by data source and setting, February 5 November 30, 2020

Healthcare Setting of Test Order	Tests Performed^**a**^ n (%)	Turnaround Time, days, Median (5th–95th Percentiles)
**Explorys**		
Outpatient/Clinic	889,802 (68.2%)	1 (0–9)
Telehealth^b^	249,918 (19.2%)	2 (0–17)
Inpatient	75,297 (5.8%)	0 (0–1)
Emergency room	67,564 (5.2%)	0 (0–3)
Long–term Care	1770 (0.1%)	10 (0–39)
Unknown	33,665 (2.6%)	1 (0–14)
**Total**	**1,303,953**	**1 (0–10)**
**Academic Health System**		
Clinic^c^	107,543 (46.3%)	1 (0–6)
Inpatient	65,131 (28.1%)	0 (0–1)
Outpatient	25,412 (11.0%)	0 (0–3)
Emergency room	24,088 (10.4%)	0 (0–4)
Observation	9836 (4.2%)	0 (0–1)
Long-term Care	20 (0.009%)	0 (0–0)
Other	5 (0.002%)	1 (0–1)
Unknown	9 (0.004%)	1 (0–23)
**Overall**	**232,044**	**0 (0–4)**
**OneFlorida**		
Outpatient/Clinic	277,108 (51.8%)	1 (0–1)
Inpatient	144,156 (27.0%)	0 (0–1)
Emergency room	55,822 (10.4%)	0 (0–1)
Observation	11,195 (2.1%)	0 (0–0)
Long-term Care	512 (0.1%)	0 (0–0)
Other	1978 (0.4%)	0 (0–0)
Unknown	46,000 (8.6%)	0 (0–1)
**Overall**	**534,525**	**1 (0–1)**

^a^single test order record can be linked to multiple encounters, and thus multiple care settings. Therefore, the sum of tests by care setting will be greater than the sum of unique tests (“Total”). The care setting(s) recorded on the associated encounter(s) linked to each test record are reflected

^b^Explorys has Telemedicine available as a separate care setting, whereas other EHR networks do not

^c^Academic Health System has the non-hospital clinic setting (“clinic”) available as a separate care setting, whereas other EHR networks combined that category with the hospital outpatient care setting

### Patient-level analyses

Data on the demographics and comorbidities of patients with interpretable NAAT results are presented in Table 2. There were 754,926, 170,926, and 358,126 unique patients with interpretable SARS-CoV-2 NAAT results in Explorys, Academic Health System, and OneFlorida, respectively. The median number of tests per patient was similar across databases, with a median (5th–95th percentile) of 1 (1–4) in Explorys, 1 (1–3) in Academic Health System, and 1 (1–3) in OneFlorida (*data not shown*).

**Table 2:** Characteristics of persons with at least one NAAT result, by EHR database, February 5–November 30, 2020

	Explorys		Academic Health System		OneFlorida	
Indicator	Patients Tested^**a**^ n (%)	Test Positivity Rate, %	Patients Tested^**a**^ n (%)	Test Positivity Rate, %	Patients Tested^**a**^ n (%)	Test Positivity Rate, %
**Total Patients**	**754,926**	**13.0**	**170,965**	**8.0**	**358,126**	**8.9**
**Age (Years)**^b^						
Mean (SD)	48.1 (21.8)	N/A	47.9 (20.8)	N/A	48.3 (22.4)	N/A
Median (IQR)	50 (31,65)	N/A	48 (31,64)	N/A	51 (31,66)	N/A
0–17	61,741 (8.2)	10.4	8879 (5.2)	5.4	32,286 (9.0)	7.4
18–44	266,189 (35.3)	15.0	70,458 (41.2)	8.1	121,398 (33.9)	11.5
45–64	225,688 (29.9)	13.8	51,724 (30.3)	8.4	108,367 (30.3)	8.7
65–74	110,972 (14.7)	10.1	22,197 (13.0)	7.3	54,394 (15.2)	6.2
75+	88,135 (11.7)	10.7	17,707 (10.4)	8.4	41,681 (11.6)	7.0
Unknown	2201 (0.3)	7.7	0 (0.0)	0.0	0 (0.0)	0.0
**Sex**^b^						
Male	324,309 (43.0)	13.4	72,236 (42.3)	8.6	158,345 (44.2)	9.5
Female	430,316 (57.0)	12.7	98,650 (57.7)	7.6	199,294 (55.6)	8.5
Unknown	301 (0.0)	9.6	79 (0.0)	3.8	487 (0.1)	3.9
**Race**^b,c^						
American Indian or Alaska Native	N/A	N/A	353 (0.2)	11.3	445 (0.1)	9.9
Asian/Pacific Islander	8250 (1.1)	14.7	2713 (1.6)	5.9	6054 (1.7)	7.0
Black/African American	182,682 (24.2)	14.7	63,085 (36.9)	10.5	58,408 (16.3)	12.2
Caucasian/ White	503,362 (66.7)	12.5	75,154 (44.0)	4.3	168,678 (47.1)	7.2
Other	18,256 (2.4)	17.7	11,732 (6.9)	18.2	86,778 (24.2)	9.8
Unknown	46,713 (6.2)	10.2	17,928 (10.5)	8.1	37,763 (10.5)	9.8
**Ethnicity**^b^						
Hispanic	39,556 (5.2)	20.7	4657 (2.7)	19.6	57,507 (16.1)	14.9
Non–Hispanic	347,228 (46.0)	13.6	147,258 (86.1)	7.2	200,823 (56.1)	7.7
Unknown	368,962 (48.9)	11.7	19,050 (11.1)	10.9	99,796 (27.9)	8.1
**Baseline Comorbidities (Charlson Comorbidity Index Component)**^d^						
*Patients with* *>* *1 comorbidity*	*267,889 (35.5)*	*11.6*	*43,763 (25.6)*	*6.9*	*82,369 (23.0)*	*6.3*
*Patients with* *>* *2 comorbidities*	*68,828 (9.1)*	*11.6*	*36,723 (21.5)*	*6.9*	*74,203 (20.7)*	*6.2*
AIDS	1595 (0.2)	11.8	1078 (0.6)	9.1	1284 (0.4)	6.2
Any Malignancy	55,176 (7.3)	8.8	8169 (4.8)	4	22,154 (6.2)	3.9
Cerebrovascul-ar Disease	33,860 (4.5)	9.7	5345 (3.1)	7.4	9095 (2.5)	6.5
Chronic Renal Failure	62,044 (8.2)	10.4	9816 (5.7)	8.5	16,245 (4.5)	7
Congestive Heart Failure	43,240 (5.7)	9.9	8947 (5.2)	7.2	15,371 (4.3)	6.3
COPD	126,099 (16.7)	11.1	15,644 (9.2)	6.3	26,214 (7.3)	6.4
Dementia	12,675 (1.7)	15.4	2436 (1.4)	12.4	4104 (1.1)	12.4
Diabetes	90,328 (12.0)	12.7	16,609 (9.7)	8.6	27,708 (7.7)	8
Diabetes w/ Sequelae	53,551 (7.1)	12.1	7336 (4.3)	8.9	13,779 (3.8)	7.4
Metastatic Solid Tumor	10,305 (1.4)	6.8	2146 (1.3)	3.1	6023 (1.7)	3.6
Moderate-Severe Liver Disease	4702 (0.6)	7.2	975 (0.6)	5.1	1984 (0.6)	3.8
Myocardial Infarction	15,737 (2.1)	9.1	3795 (2.2)	6.7	6416 (1.8)	4.9
Paralysis	6848 (0.9)	10.8	1103 (0.6)	9.5	2241 (0.6)	7.2
Peripheral Vascular Disease	47,142 (6.2)	9	8272 (4.8)	6	11,979 (3.3)	5.8
Rheumatitis	14,750 (2.0)	10.4	2044 (1.2)	5.6	3974 (1.1)	6.5
Ulcers	10,450 (1.4)	8.7	1301 (0.8)	5.5	2369 (0.7)	4.7
Various Cirrhodites	28,297 (3.7)	9.1	4822 (2.8)	5.2	10,181 (2.8)	4.9

*Acronyms*: *AIDS* acquired immunodeficiency syndrome, *COPD* chronic obstructive pulmonary disorder, *IQR* interquartile range (25th percentile, 75th percentile), *NAAT* nucleic acid amplification test, *SD* standard deviation

^a^Tested patients are those that had at least one interpretable NAAT result

^b^Measured at time of first observed NAAT in the study period

^c^American Indian or Alaska Native” is listed as ‘N/A’, not available, for Explorys because it is not a defined race category in the system. Persons in Explorys may self-identify as multiple races. Therefore, the sum of percentages may be greater than 100%

^d^CCI component categories are in alphabetical order. Comorbidities are measured in the period between −14 and − 379 days before the first observed SARS-CoV-2 NAAT in the study period. Persons may have multiple comorbidities. Therefore, the sum of percentages may be greater than 100%

The mean age of patients was 47.9–48.3 years, and the median age was 48–51 years across data sources. The majority of individuals tested were female (55.6–57.7%). Across databases, 33.9–41.2% of tested patients were between 18 and 44 years of age, and 44.0–66.7% self-identified as Caucasian/White. Results from the sensitivity analysis produced higher test positivity rates among Asian/Pacific Islander individuals in Academic Health System (6 to 12%) and in One Florida (7 to 10%) (*data not shown*).

Among tested patients, 23.0–35.5% had at least one comorbidity listed in the CCI during the baseline period of 365 days to 14 days prior to a patient’s first positive test or hospital admission. The most common comorbidities among tested patients were chronic obstructive pulmonary disease (COPD) (7.3–16.7%), diabetes (7.7–12.0%), and chronic renal failure (4.5–8.2%). The prevalence of comorbidities among test-positive patients was similar to that among patients with interpretable test results (Appendix B, Table B2).

The overall test positivity rate, by database, was 13.0% in Explorys, 8.0% in Academic Health System, and 8.9% in OneFlorida (Table 2). The test positivity rate was similar for males and females, but differed by age group, race, and ethnicity. The test positivity rate was highest among adults aged 18–44 years in Explorys and OneFlorida (15.0 and 11.5%, respectively) while, in Academic Health System, test positivity was highest among those 45–64 (8.4%) and 75 years or older (8.4%). Among patients with comorbidities, the test positivity rate was highest across databases for individuals with dementia (12.4–15.4%), diabetes (8.0–12.7%), acquired immunodeficiency syndrome (AIDS) (6.2–11.8%), paralysis (7.2–10.8%), and COPD (6.4–11.1%).

The proportion of patients hospitalized within 14 days of a positive COVID-19 NAAT result was 24.2% in Explorys, 33.1% in Academic Health System, and 26.7% in OneFlorida (Table 3). Among test-positive patients, men were hospitalized more frequently than women in all three databases. The race and ethnicity distributions for hospitalized test-positive patients (Table 3) were generally similar to those for all test positive patients (Table 2) for Explorys and OneFlorida, in that the proportions of the population represented by each race and ethnicity category did not exhibit large differences between the two cohorts. However, in Academic Health System, 48.4% of hospitalized patients were Black/African American (36.9% of all test-positive patients) in comparison to 23.9% White/Caucasian (44.0% of all test-positive patients). Those 75 years or older had the highest hospitalization rates, ranging from 46.7–69.7% across data sources. Frequently reported comorbidities observed among hospitalized test-positive patients *(data not shown)* were similar but more prevalent than among both the overall tested and test-positive populations (Appendix B, Table B2). Additional data on patient demographics, and hospitalization rates among those who tested positive are presented in Table 3.

**Table 3:** Hospitalization rates among test–positive patients by demographics, February 5–November 30, 2020

Indicator	Explorys		Academic Health System		OneFlorida	
	Patients with a Positive NAAT, n (%)	Hosp. Rate, %^**a**^	Patients with a Positive NAAT, n (%)	Hosp. Rate, %^**a**^	Patients with a Positive NAAT, n (%)	Hosp. Rate, %^**a**^
**Total Patients**	**98,436**	**24.2**	**13,650**	**33.1**	**32,025**	**26.7**
**Age (Years)**^b^						
Mean (SD)	46.4 (20.6)	N/A	48.6 (20.1)	N/A	45.0 (21.2)	N/A
Median (IQR)	46 (30,62)	N/A	48 (32,63)	N/A	44 (28,61)	N/A
0–17	6447 (6.5)	10.4	482 (3.5)	2.3	2404 (7.5)	6.4
18–44	40,031 (40.7)	17.4	5718 (41.9)	16.5	13,969 (43.6)	13.6
45–64	31,068 (31.6)	24.8	4352 (31.9)	36.8	9383 (29.3)	32.0
65–74	11,251 (11.4)	35.5	1618 (11.9)	57.5	3355 (10.5)	47.2
75+	9470 (9.6)	46.7	1480 (10.8)	69.7	2914 (9.1)	65.2
Unknown	169 (0.2)	39.6	0 (0.0)	N/A	0 (0.0)	N/A
**Sex** ^b^						
Male	43,599 (44.3)	26.6	6193 (45.4)	36.7	15,056 (47.0)	28.4
Female	54,808 (55.7)	22.3	7454 (54.6)	30.1	16,950 (52.9)	25.1
Unknown	29 (0.0)	3.4	3 (0.0)	0.0	19 (0.1)	0.0
**Race**^b,c^						
American Indian or Alaska Native	N/A	N/A	40 (0.2)	25.0	44 (0.1)	22.7
Asian/Pacific Islander	1213 (1.2)	25.9	161 (1.2)	34.2	421 (1.3)	34.2
Black/African American	26,778 (27.2)	31.6	6603 (48.4)	39.7	7145 (22.3)	34.1
Caucasian/White	63,125 (64.1)	21.5	3258 (23.9)	25.8	12,225 (38.2)	31.1
Other	3240 (3.3)	39.3	2131 (15.6)	41.5	8502 (26.5)	23.2
Unknown	4788 (4.9)	10.9	1457 (10.7)	7.3	3688 (11.5)	4.7
**Ethnicity**^b^						
Hispanic	8206 (8.3)	31.2	911 (6.7)	41.2	8568 (26.8)	30.3
Non-Hispanic	47,250 (48.0)	32.9	10,663 (78.1)	33.8	15,379 (48.0)	34.4
Unknown	43,106 (43.8)	13.4	2076 (15.2)	25.8	8078 (25.2)	8.0

*Acronyms*: *IQR* interquartile range (25th percentile, 75th percentile), *NAAT* nucleic acid amplification test, *SD* standard deviation

^a^Hospitalization rate is estimated among patients with a positive NAAT who were hospitalized within ±14 days of a positive NAAT result

^b^Measured at time of first observed NAAT in the study period

^c^“American Indian or Alaska Native” is listed as ‘N/A’, not available, for Explorys because it is not a defined race category in the system. Patients in Explorys may self-identify as multiple races. Therefore, the sum of percentages may be greater than 100%

**Fig. 1:**
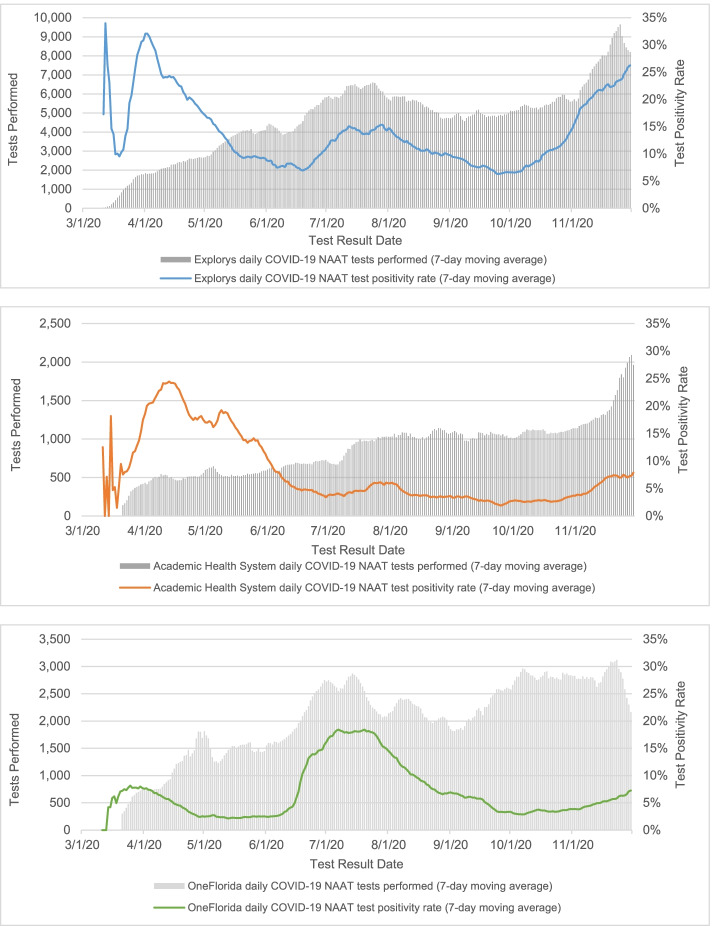
Testing volume and test positivity rate (7-day moving average) by data source, March–November 2020

Data on the temporal trends in testing volume and test positivity are presented in Fig. 1. In Explorys, the weekly testing volume (7-day moving average) peaked in July 2020 with approximately 6500 tests conducted per week, and again in November 2020 (over 9000 weekly tests). In Academic Health System, the testing volume only peaked in November 2020 (over 2000 weekly tests). Testing volumes fluctuated throughout the study period in OneFlorida, with one peak in July 2020 (2700 weekly tests) and another in November 2020 (2500 weekly tests).

Temporal trends in test positivity rates followed similar patterns in both Explorys and Academic Health System although the rate was higher in Explorys. In both systems, the test positivity rate peaked in early-to-mid-March (20–35%), then dipped before steadily increasing again to produce a second peak in early-to-mid-April (25–35%). As testing volumes increased into the summer months, the test positivity rates in both Explorys and Academic Health System decreased steadily until the end of June 2020 (reaching a low of 2–7%). A third, smaller peak, occurred in mid-to-late July (12–18% in Explorys; 5–10% in Academic Health System), followed by another decrease until the fall, where positivity rates began to rise again and reached a fourth peak at the end of November (26% in Explorys; 8% in Academic Health System). Conversely, in OneFlorida, the test positivity rate only reached 8% in early March, dropping to below 4% until the beginning of June, when it began to rise, reaching a peak of 18–20% in the middle of July. The test positivity rates in OneFlorida decreased, hovering below 5% in the fall months before rising again, similar to Explorys and Academic Health System, to 7% in November.

**Fig. 2:**
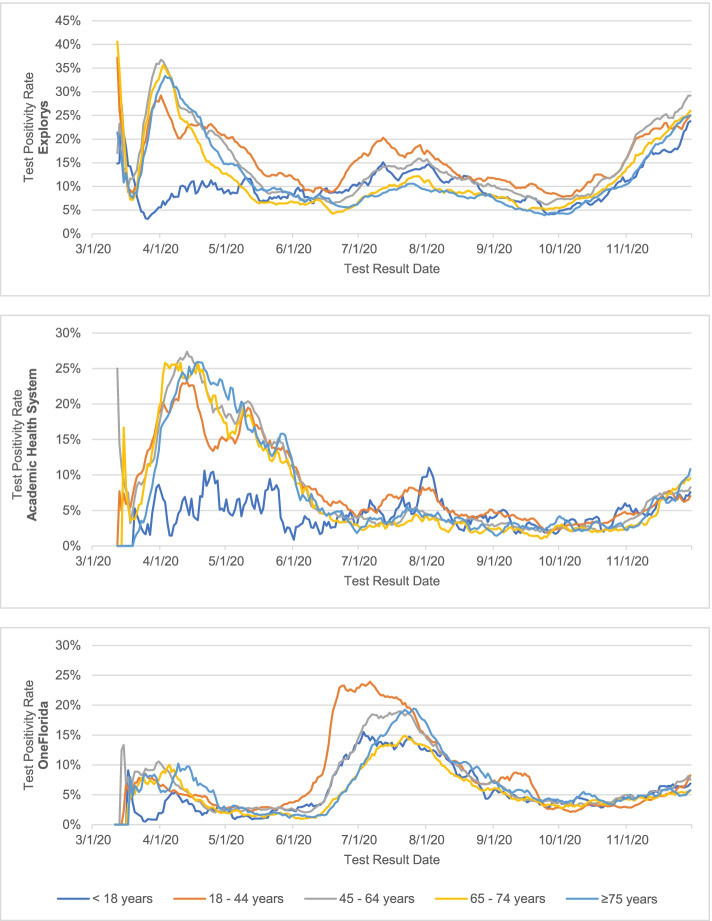
Test positivity rate (7-day moving average), by age group and data source, March–November 2020

Data on age-specific test positivity trends are presented in Fig. 2. Starting in April 2020, the test positivity trends by age group followed similar trends in both Explorys and Academic Health System. Test positivity peaked in mid-April 2020 for individuals 18–44 (25–30%), 45–64 (27–35%), 65–74 (25–35%), and 75 years and older (27–35%). This was substantially higher than the test positivity rate among those under the age of 18 during the same time period (2–11%). In OneFlorida, test positivity peaked at the end of June or early July 2020 for all five age groups, reaching 15% among those younger than 18 years old, 24% among those 18–44 years old, 19% among those 45–64 years old, 15% among those 65–74 years old, and 19% among those 75 years and older.

## Discussion

This study provides a descriptive analysis of the testing patterns, test positivity rate, and demographic and clinical characteristics of patients tested and hospitalized from over 1.8 million COVID-19 NAATs, representing 1.1 million people from three large EHR systems serving patients across six states in an eastern region of the U.S. Such findings help improve understanding of risk factors for receiving a positive COVID-19 test result in the pre-vaccination era. This study also demonstrates the feasibility of utilizing NAAT-positive results that confirm the COVID-19 testing status of patients across different EHRs, which could be used as components of EHR-based cohort definitions that depend on COVID-19 testing results of patients.

During the first year of the COVID-19 pandemic, test positivity rates increased starting in April 2020 and peaked in July (OneFlorida) and November (Explorys and Academic Health System), mirroring the progression of the first wave of the pandemic in the eastern U.S. For example, the John’s Hopkins Coronavirus Resource Center [8], which tracks test positivity by state and region, observed a similar test positivity peak in July 2020 for Florida (12%). Test positivity rates also demonstrated a marked peak in April 2020 for Explorys and the Academic Health System; while this may have been due in part to low testing volume, such a peak was not observed in the OneFlorida data, suggesting that early transmission may have been lower in Florida compared to some other states.

The overall test positivity rate ranged between 8 and 13%, varying by data source, age group, and ethnicity. It should be noted that the characteristics of the population covered — including population size, pre-existing comorbidities, and age, race, and ethnic distributions — differed across database, as detailed in Table 2. Differences in the geographic coverage of the three EHR systems, such as the varying prevalence of the disease and availability of test kits can explain some of the variation in the test positivity rates. Increasing availability, demand, and use of diagnostic testing is likely to capture a more accurate estimate of actual transmission patterns in communities, as lower testing volume can lead to an overestimation of the positivity rate due to disproportionate testing of individuals most likely to be infected. Explorys contained data on health encounters predominantly from across five eastern U.S. states (Ohio, Louisiana, Georgia, Florida, and New York), while Academic Health System encounters were primarily from the Mid-Atlantic region, and OneFlorida only contained data on health encounters from Florida. All three databases demonstrated a peak in testing volume in July 2020, with an additional peak in November observed in Explorys and Academic Health System. These patterns are generally consistent with those observed by The COVID-19 Tracking Project [9] — which observed peaks in national testing volume in mid-July and late November — and is likely due to a combination of increasing testing availability and demand as new waves of transmission emerged. However, despite increases in demand, the median turnaround time from test order to result did not exceed 2 days in any month across the three databases.

There was a median of one test per patient observed in all three databases. We found the highest test positivity rate among those 18–44 years of age across the three data sources. This is consistent with data from the U.S. Centers for Disease Control and Prevention, which reported a consistently higher test positivity rate in those 18–24 and 25–45 years of age across regions of the U.S. between June and July 2020 [10]. Although test positivity rates in some studies of eastern U.S. patient cohorts have been shown to increase with age [11–15], another study from Martinez and colleagues, reported higher test positivity rates among younger individuals (18–44 years old) in a large cohort study of over 6000 COVID-19 patients from the Johns Hopkins Healthcare System (covering the Mid-Atlantic region similar to our study) [16]. Unlike other large population-based studies, differences in the test positivity rate by sex were not observed in the present study [11, 12, 15, 17]. However, it should be noted that all of these studies showing test positivity differences by sex only assessed patients identified between March 2020 and June 2020, meaning that shifts in test positivity rates throughout the pandemic would not have been captured whereas our study period continued through November 2020 [11–15, 17]. Other design differences across studies, particularly geographic coverage and study populations, could also have led to differences in test positivity estimates.

Data suggest that those self-identifying as Black/African American had a 2–6% higher test positivity rate than those self-identifying as White/Caucasian, while those self-identifying as Hispanic had a 7–12% higher test positivity rate compared to non-Hispanic. There is substantial epidemiological evidence that Black/African American and Hispanic populations are disproportionately affected by COVID-19 [12, 15, 16, 18–26]. Several factors have been identified which are likely to contribute to this trend, including socio-economic status which impacts the ability to practice social distancing, household size, population density, differences in healthcare-seeking behaviors, access to testing, lower rates of insurance coverage, and higher prevalence of comorbidities associated with COVID-19 (such as obesity) [15, 16, 18, 24]. However, incomplete reporting of race and ethnicity data remains a challenge for refining our understanding of race- and ethnicity-based risk factors for COVID-19 infection and severe outcomes. In the present study, for example, race was unknown for 6.2–9.8% of patients with an interpretable NAAT, and ethnicity was unknown for 8.1–11.7% across three data sources. A sensitivity analysis (quantitative bias analysis) was conducted to assess the potential impact of this missingness, with increases observed in test positivity rates among Asian/Pacific Islander individuals. The rates of hospitalization among those who self-identified as Black/African American remained consistently higher than among White/Caucasians, while hospitalization rates were fairly similar between those self-identifying as Hispanic/Latino and non-Hispanic.

One quarter to one third of all test-positive patients were hospitalized within 14 days of their positive test result. Those over the age of 75 had the highest hospitalization rates (47–70%). Over two thirds of test-positive patients over the age of 45 were hospitalized across the three databases. Among test-positive patients, men were hospitalized more frequently than women across all three databases, while no single race or ethnicity category stood out as having the highest hospitalization rate across all three databases. However, we found that test-positive patients identifying as Black/African American had higher hospitalization rates compared to test-positive patients identifying as White/Caucasian in two out of three databases (Explorys and Academic Health System). This is consistent with similar studies that also found higher hospitalization rates among Black/African American individuals compared to White/Caucasian test-positive patients [12–14, 27–30]. Notably, two large studies from New York and Houston found that, although non-Hispanic Black/African American and Hispanic/Latino individuals were more likely to be hospitalized, after adjusting for demographics and other factors such as vital signs, there was a non-significantly lower risk of in-hospital mortality among Black/African American and Hispanic patients than among White/Caucasian individuals [12, 30]. Nevertheless, our study did not evaluate in-hospital mortality.

The strengths of this study include providing insight into the characteristics of those receiving COVID-19 tests and positive test results in the early stages of the pandemic. Higher positivity and hospitalization rates in some groups suggests higher disease circulation and severe outcomes in at-risk populations and contributes to the understanding of COVID-19 risk factors. Few studies have conducted similar longitudinal analyses of such a large and geographically distributed patient cohort. The broader geographic scope and longer time horizon enabled by the FDA BEST Initiative network supports improved geographic representativeness of the patient cohort, enhancing the external validity and generalizability of findings. This demonstrates the value of the BEST Initiative network’s capabilities, which can be utilized to conduct active surveillance of biologic products and other public health issues under the purview of FDA.

This study has some limitations that should be considered when interpreting the results. First, testing practices differed across states and changed over time within each geographic region included in this analysis, which suggests positivity estimates could have been impacted by factors other than infection transmission patterns. The United States does not have a single, national healthcare or public health system, and practices can vary substantially across states. For example, states had differential access to diagnostic tests and took different approaches to testing [31]. Early on, many states restricted testing to individuals who had travelled to high-risk countries or had a known close contact (usually defined as being within 6 ft of a case for a cumulative total of 15 minutes over a 24-hour period) [32]. While test supplies increased over time, some states were also met with high demand due to increases in transmission, which required some health departments to implement restrictions on testing, including the severity of symptoms, contact with a case, or risk status [31, 32]. Second, the study population, while large and distributed, was concentrated in a region of the eastern U.S. and not geographically representative of the entire U.S. population. Third, we were unable to calculate rates of testing among the overall EHR populations, due to gaps in observability within the EHR databases for tests that may be conducted outside of the EHR system’s catchment. For the same reason, there is a risk that COVID-19-positive patients were misclassified as negative since tests conducted outside of the healthcare systems would not have been captured. The testing patterns within these EHR systems may also differ meaningfully from those with tests conducted outside these EHR systems which could impact the generalizability of our findings. Lastly, analyses of the hospitalized population were unable to differentiate between individuals who were hospitalized due to COVID-19 infection and those with an incidental infection unrelated to their reason for admission. Future studies may be needed to further evaluate testing volume, test positivity rate and hospitalization among test-positive patients in other healthcare systems and regions of the U.S. during the same phase of the pandemic.

## Supplementary Information

**Additional file 1: Appendix A.** LOINC Codes for Identification of SARS-CoV-2 NAA Diagnostics, February 5–November 30, 2020.

**Additional file 2: Appendix B.** Supplementary Analyses.

## Data Availability

All data generated or analysed during this study are included in this published article and its supplementary information files.
