# Supplementary material for: Characterization of test positivity among patients with coronavirus disease 2019 (COVID-19) in three electronic health records databases, February–November 2020
Source: BMC Public Health. 2022 Jun 18;22:1217. doi: 10.1186/s12889-022-13635-6 (PMC9206507; doi:10.1186/s12889-022-13635-6)
Supplement: Supplementary file 1 — Additional file 1: Appendix A. LOINC Codes for Identification of SARS-CoV-2 NAA Diagnostics, February 5–November 30, 2020. [file 12889_2022_13635_MOESM1_ESM.docx]

**Appendix A. LOINC Codes for Identification of SARS-CoV-2 NAA Diagnostics, February 5–November 30, 2020**

| **LOINC** | **Long Common Name** |
| --- | --- |
| 94745-7 | SARS-CoV-2 (COVID-19) RNA [Cycle Threshold #] in Respiratory specimen by NAA with probe detection |
| 94746-5 | SARS-CoV-2 (COVID-19) RNA [Cycle Threshold #] in Unspecified specimen by NAA with probe detection |
| 94819-0 | SARS-CoV-2 (COVID-19) RNA [Log #/volume] (viral load) in Unspecified specimen by NAA with probe detection |
| 94565-9 | SARS coronavirus 2 RNA [Presence] in Nasopharynx by NAA with non-probe detection |
| 94759-8 | SARS-CoV-2 (COVID-19) RNA [Presence] in Nasopharynx by NAA with probe detection |
| 94500-6 | SARS coronavirus 2 RNA [Presence] in Respiratory specimen by NAA with probe detection |
| 94845-5 | SARS-CoV-2 (COVID-19) RNA [Presence] in Saliva (oral fluid) by NAA with probe detection |
| 94660-8 | SARS-CoV-2 (COVID-19) RNA [Presence] in Serum or Plasma by NAA with probe detection |
| 94309-2 | SARS Coronavirus 2 RNA [Presence] in Unspecified specimen Qualitative by NAA with probe detection |
| 41458-1 | SARS coronavirus RNA [Presence] in Unspecified specimen by NAA with probe detection |
| 94534-5 | SARS coronavirus 2 RdRp gene [Presence] in Respiratory specimen by NAA with probe detection |
| 95608-6 | SARS-CoV-2 (COVID-19) RNA [Presence] in Respiratory specimen by NAA with non-probe detection |
| 94533-7 | SARS-CoV-2 (COVID19) N gene [Presence] in Respiratory specimen by NAA with probe detection |
| 94640-0 | SARS coronavirus 2 S gene [Presence] in Respiratory specimen by NAA with probe detection |
| 94559-2 | SARS coronavirus 2 ORF1ab region [Presence] in Respiratory specimen by NAA with probe detection |
| 94502-2 | SARS-related coronavirus RNA [Presence] in Respiratory specimen by NAA with probe detection |
| 95423-0 | Influenza virus A + B and SARS-CoV-2 (COVID-19) identified in Respiratory specimen by NAA with probe detection |
| 95409-9 | SARS coronavirus 2 (COVID19) N gene [Presence] in Nose by NAA with probe detection |
| 95425-5 | SARS-CoV-2 (COVID-19) N gene [Presence] in Saliva (oral fluid) by NAA with probe detection |
| 94760-6 | SARS coronavirus 2 N gene [Presence] in Nasopharynx by NAA with probe detection |
| 95406-5 | SARS-CoV-2 (COVID19) RNA [Presence] in Nose by NAA with probe detection |
| 94758-0 | SARS-related coronavirus E gene [Presence] in Respiratory specimen by NAA with probe detection |
| 96091-4 | SARS-CoV-2 (COVID-19) RdRp gene [Presence] in Saliva (oral fluid) by NAA with probe detection |
| 94316-7 | SARS-CoV-2 (COVID-19) N gene [Presence] in Specimen by NAA with probe detection |

Source: Centers for Diseases Control and Prevention. LOINC In Vitro Diagnostic (LIVD) Test Code Mapping for SARS-CoV-2 Tests. (Accessed April 1, 2021, at <https://www.cdc.gov/csels/dls/sars-cov-2-livd-codes.html>.)
