# Supplementary material for: Characterization of test positivity among patients with coronavirus disease 2019 (COVID-19) in three electronic health records databases, February–November 2020
Source: BMC Public Health. 2022 Jun 18;22:1217. doi: 10.1186/s12889-022-13635-6 (PMC9206507; doi:10.1186/s12889-022-13635-6)
Supplement: Supplementary file 2 — Additional file 2: Appendix B. Supplementary Analyses. [file 12889_2022_13635_MOESM2_ESM.docx]

**Appendix B. Supplementary Analyses**

**Table B1.** Distribution of number of tests and median days between test order and result by data source and month, February 5–November 30, 2020

|  | Explorys^a^ | | Academic Health System | | OneFlorida | |
| --- | --- | --- | --- | --- | --- | --- |
| Month | **Tests Performed, n** | **Turnaround Time, days, Median (5^th^–95^th^ Percentiles)** | **Tests Performed, n** | **Turnaround Time, days, Median (5^th^–95^th^ Percentiles)** | **Tests Performed, n** | **Turnaround Time, days, Median (5^th^–95^th^ Percentiles)** |
| Overall^a^ | **830,182** | **1 (0–10)** | **232,044** | **0 (0–4)** | **534,525** | **1 (0–1)** |
| March | 17,411 | 2 (0–11) | 4,935 | 2 (0–10) | 8,861 | 1 (0–2) |
| April | 58,399 | 1 (0–12) | 15,562 | 0 (0–6) | 36,824 | 1 (0–1) |
| May | 93,014 | 1 (0–7) | 17,199 | 0 (0–2) | 44,495 | 1 (0–2) |
| June | 103,972 | 1 (0–7) | 20,565 | 0 (0–3) | 64,154 | 1 (0–1) |
| July | 138,247 | 1 (0–8) | 28,606 | 0 (0–5) | 76,435 | 1 (0–1) |
| August | 116,968 | 1 (0–8) | 33,038 | 1 (0–3) | 65,665 | 0 (0–1) |
| September | 96,009 | 1 (0–13) | 31,512 | 0 (0–3) | 68,712 | 1 (0–1) |
| October | 98,053 | 1 (0–13) | 34,030 | 0 (0–3) | 88,089 | 1 (0–1) |
| November | 108,079 | 1 (0–14) | 46,597 | 1 (0–4) | 81,290 | 1 (0–1) |
| ^a^ Measures in this table referencing test orders in Explorys are based on 830,182 out of 1,303,953 total interpretable test results that have a test order found in the preceding 45 days of a test result. This is due to test result and test order records not being directly linked at the test-level in Explorys. | | | | | | |

**Table B2.** Demographics, comorbidities for (1) Care-seeking patients who had at least one healthcare encounter, but no observable COVID-19 NAAT, (2) Patients with at least one non–null NAAT, and (3) Patients with positive COVID-19 NAAT, by EHR database, February 5–November 30, 2020

|  | **Explorys** | | | **Academic Health System** | | | **OneFlorida** | | |
| --- | --- | --- | --- | --- | --- | --- | --- | --- | --- |
| **Indicator** | **Care-seeking Patients with no Observable NAAT,^a^**  **n (%)** | **Patients Tested,^b^**  **n (%)** | **Patients with positive NAAT,**  **n (%)** | **Care-seeking Patients with no Observable NAAT,^a^**  **n (%)** | **Patients Tested,^b^**  **n (%)** | **Patients with Positive NAAT,**  **n (%)** | **Care-seeking Patients with no Observable NAAT,^a^**  **n (%)** | **Patients Tested,^b^**  **n (%)** | **Patients with Positive NAAT,**  **n (%)** |
| **Total Patients** | **7,368,895** | **754,926** | **98,436** | **786,049** | **170,965** | **13,650** | **1,790,074** | **358,126** | **32,025** |
| **Age (Years)**^c^ |  |  |  |  |  |  |  |  |  |
| Mean (SD) | 41.6 (25.4) | 48.1 (21.8) | 46.4 (20.6) | 50.2 (22.2) | 47.9 (20.8) | 48.6 (20.1) | 40.7 (24.7) | 48.3 (22.4) | 45.0 (21.2) |
| Median (IQR) | 42 (18,63) | 50 (31,65) | 46 (30,62) | 53 (33,68) | 48 (31,64) | 48 (32,63) | 41 (20,61) | 51 (31,66) | 44 (28,61) |
| 0-17 | 1,786,656 (24.2) | 61,741 (8.2) | 6,447 (6.5) | 82,941 (10.6) | 8,879 (5.2) | 482 (3.5) | 390,202 (21.8) | 32,286 (9.0) | 2,404 (7.5) |
| 18-44 | 2,017,626 (27.4) | 266,189 (35.3) | 40,031 (40.7) | 287,365 (36.6) | 70,458 (41.2) | 5,718 (41.9) | 582,350 (32.5) | 121,398 (33.9) | 13,969 (43.6) |
| 45-64 | 1,782,731 (24.2) | 225,688 (29.9) | 31,068 (31.6) | 229,017 (29.1) | 51,724 (30.3) | 4,352 (31.9) | 449,730 (25.1) | 108,367 (30.3) | 9,383 (29.3) |
| 65-74 | 968,311 (13.1) | 110,972 (14.7) | 11,251 (11.4) | 104,910 (13.3) | 22,197 (13.0) | 1,618 (11.9) | 208,443 (11.6) | 54,394 (15.2) | 3,355 (10.5) |
| 75+ | 748,067 (10.2) | 88,135 (11.7) | 9,470 (9.6) | 81,788 (10.4) | 17,707 (10.4) | 1,480 (10.8) | 159,235 (8.9) | 41,681 (11.6) | 2,914 (9.1) |
| Unknown | 65,504 (0.9) | 2,201 (0.3) | 169 (0.2) | 28 (0.0) | 0 (0.0) | 0 (0.0) | 114 (0.0) | 0 (0.0) | 0 (0.0) |
| **Sex**^c^ |  |  |  |  |  |  |  |  |  |
| Male | 3,236,256 (43.9) | 324,309 (43.0) | 43,599 (44.3) | 324,685 (41.3) | 72,236 (42.3) | 6,193 (45.4) | 768,101 (42.9) | 158,345 (44.2) | 15,056 (47.0) |
| Female | 4,123,783 (56.0) | 430,316 (57.0) | 54,808 (55.7) | 460,912 (58.6) | 98,650 (57.7) | 7,454 (54.6) | 1,021,034 (57.0) | 199,294 (55.6) | 16,950 (52.9) |
| Unknown | 8,856 (0.1) | 301 (0.0) | 29 (0.0) | 452 (0.1) | 79 (0.0) | 3 (0.0) | 939 (0.1) | 487 (0.1) | 19 (0.1) |
| **Race**^c,d^ |  |  |  |  |  |  |  |  |  |
| American Indian or Alaska Native | N/A | N/A | N/A | 1,883 (0.2) | 353 (0.2) | 40 (0.2) | 2,895 (0.2) | 445 (0.1) | 44 (0.1) |
| Asian/Pacific Islander | 80,113 (1.1) | 8,250 (1.1) | 1,213 (1.2) | 13,143 (1.7) | 2,713 (1.6) | 161 (1.2) | 35,347 (2.0) | 6,054 (1.7) | 421 (1.3) |
| Black/African American | 993,296 (13.5) | 182,682 (24.2) | 26,778 (27.2) | 243,826 (31.0) | 63,085 (36.9) | 6,603 (48.4) | 322,798 (18.0) | 58,408 (16.3) | 7,145 (22.3) |
| Caucasian/White | 3,932,780 (53.4) | 503,362 (66.7) | 63,125 (64.1) | 362,887 (46.2) | 75,154 (44.0) | 3,258 (23.9) | 1,043,989 (58.3) | 168,678 (47.1) | 12,225 (38.2) |
| Other | 195,401 (2.7) | 18,256 (2.4) | 3,240 (3.3) | 51,231 (6.5) | 11,732 (6.9) | 2,131 (15.6) | 265,708 (14.8) | 86,778 (24.2) | 8,502 (26.5) |
| Unknown | 2,209,191 (30.0) | 46,713 (6.2) | 4,788 (4.9) | 113,079 (14.4) | 17,928 (10.5) | 1,457 (10.7) | 119,337 (6.7) | 37,763 (10.5) | 3,688 (11.5) |
| **Ethnicity**^c^ |  |  |  |  |  |  |  |  |  |
| Hispanic | 211,386 (2.9) | 39,556 (5.2) | 8,206 (8.3) | 19,427 (2.5) | 4,657 (2.7) | 911 (6.7) | 455,510 (25.4) | 57,507 (16.1) | 8,568 (26.8) |
| Non-Hispanic | 3,454,743 (46.9) | 347,228 (46) | 47,250 (48.0) | 649,046 (82.6) | 147,258 (86.1) | 10,663 (78.1) | 1,193,756 (66.7) | 200,823 (56.1) | 15,379 (48.0) |
| Unknown | 3,708,160 (50.3) | 368,962 (48.9) | 43,106 (43.8) | 117,576 (15.0) | 19,050 (11.1) | 2,076 (15.2) | 140,808 (7.9) | 99,796 (27.9) | 8,078 (25.2) |
| **Baseline Comorbidities (Charlson Comorbidity Index Component)**^e^ | | | | | | | | | |
| Patients with >1 comorbidity | 1,233,505 (16.7) | 267,889 (35.5) | 30,996 (31.5) | 127,186 (16.2) | 43,763 (25.6) | 3,035 (22.2) | 361,226 (20.2) | 82,369 (23.0) | 5,167 (16.1) |
| Patients with >2 comorbidities | 1,076,027 (14.6) | 68,828 (9.1) | 8,000 (8.1) | 78,830 (10.0) | 36,723 (21.5) | 2,539 (18.6) | 282,013 (15.8) | 74,203 (20.7) | 4,622 (14.4) |
| AIDS | 6,963 (0.1) | 1,595 (0.2) | 188 (0.2) | 2,216 (0.3) | 1,078 (0.6) | 98 (0.7) | 4,819 (0.3) | 1,284 (0.4) | 79 (0.2) |
| Any Malignancy | 162,452 (2.2) | 55,176 (7.3) | 4,878 (5.0) | 17,797 (2.3) | 8,169 (4.8) | 327 (2.4) | 72,469 (4.0) | 22,154 (6.2) | 869 (2.7) |
| Cerebrovascular Disease | 92,035 (1.2) | 33,860 (4.5) | 3,272 (3.3) | 12,261 (1.6) | 5,345 (3.1) | 398 (2.9) | 26,267 (1.5) | 9,095 (2.5) | 590 (1.8) |
| Chronic Renal Failure | 161,373 (2.2) | 62,044 (8.2) | 6,466 (6.6) | 15,334 (2.0) | 9,816 (5.7) | 834 (6.1) | 40,635 (2.3) | 16,245 (4.5) | 1,142 (3.6) |
| Congestive Heart Failure | 122,090 (1.7) | 43,240 (5.7) | 4,302 (4.4) | 13,816 (1.8) | 8,947 (5.2) | 641 (4.7) | 33,614 (1.9) | 15,371 (4.3) | 973 (3.0) |
| COPD | 778,125 (10.6) | 126,099 (16.7) | 14,044 (14.3) | 31,367 (4.0) | 15,644 (9.2) | 985 (7.2) | 90,432 (5.1) | 26,214 (7.3) | 1,690 (5.3) |
| Dementia | 32,822 (0.4) | 12,675 (1.7) | 1,947 (2.0) | 3,850 (0.5) | 2,436 (1.4) | 303 (2.2) | 10,443 (0.6) | 4,104 (1.1) | 507 (1.6) |
| Diabetes | 313,231 (4.3) | 90,328 (12.0) | 11,480 (11.7) | 45,883 (5.8) | 16,609 (9.7) | 1,430 (10.5) | 116,094 (6.5) | 27,708 (7.7) | 2,210 (6.9) |
| Diabetes w/ Sequelae | 149,157 (2.0) | 53,551 (7.1) | 6,495 (6.6) | 8,390 (1.1) | 7,336 (4.3) | 653 (4.8) | 37,150 (2.1) | 13,779 (3.8) | 1,016 (3.2) |
| Metastatic Solid Tumor | 25,395 (0.3) | 10,305 (1.4) | 701 (0.7) | 2,649 (0.3) | 2,146 (1.3) | 66 (0.5) | 11,560 (0.6) | 6,023 (1.7) | 214 (0.7) |
| Moderate-Severe Liver Disease | 7,663 (0.1) | 4,702 (0.6) | 339 (0.3) | 741 (0.1) | 975 (0.6) | 50 (0.4) | 3,226 (0.2) | 1,984 (0.6) | 76 (0.2) |
| Myocardial Infarction | 40,566 (0.6) | 15,737 (2.1) | 1,425 (1.4) | 3,913 (0.5) | 3,795 (2.2) | 253 (1.9) | 13,215 (0.7) | 6,416 (1.8) | 317 (1.0) |
| Paralysis | 18,087 (0.2) | 6,848 (0.9) | 741 (0.8) | 1,716 (0.2) | 1,103 (0.6) | 105 (0.8) | 5,198 (0.3) | 2,241 (0.6) | 162 (0.5) |
| Peripheral Vascular Disease | 121,011 (1.6) | 47,142 (6.2) | 4,252 (4.3) | 14,337 (1.8) | 8,272 (4.8) | 496 (3.6) | 29,154 (1.6) | 11,979 (3.3) | 697 (2.2) |
| Rheumatitis | 48,465 (0.7) | 14,750 (2.0) | 1,534 (1.6) | 5,522 (0.7) | 2,044 (1.2) | 115 (0.8) | 18,079 (1.0) | 3,974 (1.1) | 259 (0.8) |
| Ulcers | 24,706 (0.3) | 10,450 (1.4) | 910 (0.9) | 853 (0.1) | 1,301 (0.8) | 72 (0.5) | 3,628 (0.2) | 2,369 (0.7) | 112 (0.3) |
| Various Cirrhodites | 74,246 (1.0) | 28,297 (3.7) | 2,573 (2.6) | 7,635 (1.0) | 4,822 (2.8) | 250 (1.8) | 28,271 (1.6) | 10,181 (2.8) | 499 (1.6) |
| Acronyms: AIDS: acquired immunodeficiency syndrome, COPD: chronic obstructive pulmonary disorder, IQR: interquartile range (25th percentile, 75th percentile) NAAT: nucleic acid amplification test, SD: standard deviation  **Notes:**  ^a^ Care-seeking patients are those that had at least one health encounter in the database.  ^b^ Tested patients are those that had at least one interpretable NAAT result.  ^c^ Measured at time of first observed NAAT in the study period.  ^d^ American Indian or Alaska Native” is listed as ‘N/A’, not available, for Explorys because it is not a defined race category in the system. Persons in Explorys may self-identify as multiple races. Therefore, the sum of percentages may be greater than 100%.  ^e^ CCI component categories are in alphabetical order. Comorbidities are measured in the period between -14 and -379 days before the first observed SARS-CoV-2 NAAT in the study period. Persons may have multiple comorbidities. Therefore, the sum of percentages may be greater than 100%. | | | | | | | | | |
